# Necrotizing Soft Tissue Fasciitis after Intramuscular Injection

**DOI:** 10.1155/2018/3945497

**Published:** 2018-05-08

**Authors:** Angelica Abbate, Piero Luigi Almasio, Martina Mongitore, Gaetano Di Vita, Rosalia Patti

**Affiliations:** ^1^General Surgery Unit, Department of Surgical, Oncological and Stomatological Sciences, University of Palermo, Palermo, Italy; ^2^Biomedical Department of Internal and Specialistic Medicine, University of Palermo, Palermo, Italy

## Abstract

Necrotizing soft tissue fasciitis (NSTIs) or necrotizing fasciitis is an infrequent and serious infection. Herein, we describe the clinical course of a female patient who received a diagnosis of NSTIs after gluteus intramuscular injection. We also report the results of our review of published papers from 1997 to 2017. Since now, 19 cases of NSTIs following intramuscular injections have been described. We focus on the correlation between intramuscular injection and NSTIs onset, especially in immunosuppressed patients treated with corticosteroids, suffering from chronic diseases or drug addicted. Intramuscular injections can provoke severe tissue trauma, representing local portal of infection, even if correctly administrated. Otherwise, it is important not to inject drug in subcutaneous, which is a less vascularized area and therefore more susceptible to infections. Likewise, a proper injecting technique and aspiration prior to injection seem to be valid measure to prevent intra-arterial or para-arterial drug injection with the consequent massive inflammatory reaction. Necrosis at the infection site appears to be independent of the drug, and it is a strong additional risk factor for NSTIs.

## 1. Introduction

Necrotizing soft tissue fasciitis (NSTIs) or necrotizing fasciitis is a rare and severe infection characterized by rapid and extensive necrosis of the dermis, subcutaneous tissue, fat, superficial fascia, deep fascia, or muscle, associated with a high mortality rate.

The infection is caused by mono- or polymicrobial flora of both anaerobic and aerobic germs. The common etiologies of NSTIs are insect bites, chronic wounds, trauma, or idiopathic origin. Otherwise, possible and infrequent sources are iatrogenic, for example, injections or surgical wounds.

Several small studies and cases reports have been published describing fulminant NSTIs after injection. Only a case study of 10 patients with NSTIs after intramuscular injections has been reported in a recent work [1].

## 2. Case Report

A 60-year-old woman, suffering since 10 years from rheumatoid arthritis and treated since one month with nonsteroidal anti-inflammatory drugs (NSAIDs) (Diclofenac 75 mg, 2 tablets a day) and corticosteroids (methylprednisolone 16 mg, 1 tablet a day), was admitted to our department after the occurrence of a large bulla on her left gluteus.

Her relatives referred an intramuscular injection of ceftriaxone in the same site 8 days before for an upper respiratory tract infection. They also referred severe pain onset at the site of injection 24 hours later, which seemed out of proportion to physical findings and the appearance of a bulla after 3 days which rapidly grown up to the size of 8 × 10 centimeters. They reported that she had been sleepy and confused since 2 days before.

At admission, the patient showed poor physical conditions and pallor; she was in forced supine decubitus, in drowsiness state, and disoriented. Vital signs were as follows: skin temperature 37.2°C, blood pressure 80/60 mmHg, hearth rate 110 bpm, and SpO_2_ between 86% and 96% (at a flow rate of 4 ml per minute). Arterial blood gas analysis was performed, revealing altered values: pH 7.32, pCO_2_ 41.1 mmHg, and pO_2_ 49.1 mmHg. Blood tests revealed increased leukocyte count of 16.15 × 10^3^/*μ*l and neutrophil count 14.99 × 10^3^/*μ*l. The following values were also noted: serum protein, 4.6 g/dl; albumin, 1.7 g/dl; and C-reactive protein, 50 mg/dl.

At physical examination, she looked dyspnoic with subcutaneous emphysema, bullae, ecchymosis, blisters, gaseous gangrene, and skin sloughing involving neck bilaterally, left thorax wall, and left abdominal wall up to the root of the left thigh.

**Figure 1:**
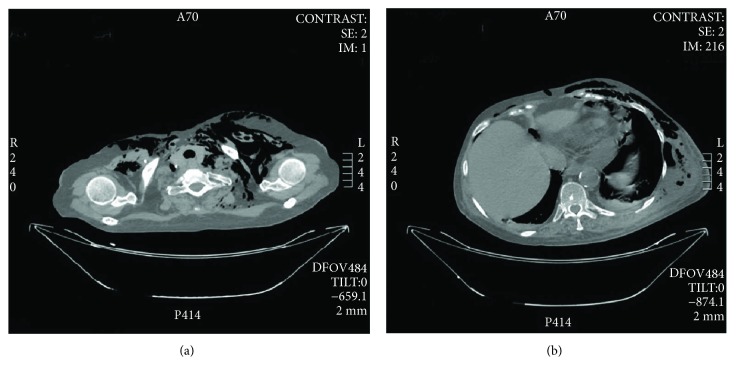


**Figure 2:**
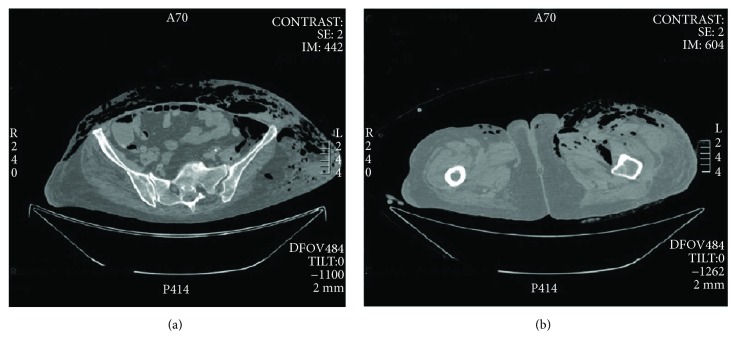
(a) Abdominal CT scan detecting intraperitoneal free air. (b) Subcutaneous emphysema of the left thigh root.

CT scan confirmed left laterocervical subcutaneous emphysema (Figure 1(a)), the presence of fluid collection with air-fluid level in left thorax-abdominal subcutaneous soft tissues (Figure 1(b)), subcutaneous emphysema of the left thigh root (Figure 2(b)), revealing further pneumomediastinum, intraperitoneal free air (Figure 2(a)), left femoral vein thrombosis and thromboembolism of the right pulmonary artery. Antibiotic therapy with teicoplanin 1200 mg once a day, metronidazole 500 mg four times a day, imipenem/cilastatin 500 mg four times a day, and caspofungin 70 mg once a day was planned according to an infettivologist suggestion.

She received a prompt treatment with radical surgical debridement and resection of the necrotic tissue, and, few hours later, she underwent hyperbaric chamber treatment.

The culture test resulted positive to *Proteus mirabilis*, *Acinetobacter baumannii*, *Candida albicans*, and *Escherichia coli*; the histological examination of the tissue removed from the left hip described a colliquative necrosis, associated with widespread infiltration of granulocytes, abscess-like.

Twenty-four hours later, she received another surgical debridement, with further removal of necrotic tissue. Nevertheless, she had a rapid clinical deterioration with multiple organ failure and she died two days after the admission.

## 3. Discussion

From the review of literature, only 19 cases of NSTIs after intramuscular injections have been reported. All these are case reports except one [1], in which the authors describe 10 cases of NSTIs after intramuscular injection observed over a period of 10 years; otherwise, it is hard to extract patients' data because the revision includes also cases of NSTIs after infiltrations.

In our review, the average age is 57 years ranging from 24 to 83 years and the majority of the patients are women. We deal especially with immunosuppressed patients, drug addicts, or diabetics. The primary site of intramuscular injection was above all the buttock, and the necrosis appeared always in the same site of injection. In 9 cases, it was possible to identify the drug injected. In 6 of these cases, it was NSAIDs; only one case was after antibiotic injection, another one after corticosteroid and mepivacaine injection [2], and another after “bath salt” injection.

It becomes known that the average space of time between intramuscular injection and surgical treatment is 4 days, but in all cases, surgery was performed on the same day of admission. Therefore, we can suppose that in all cases with a period longer than 4 days, the delayed hospital admission is to attribute to not aggressive early manifestation of NSTIs.

Examining these case reports, it comes out that a clinical trial of pain out of proportion, swelling, and fever is usually present in patients affected by NSTIs, in addiction to tenderness and erythema. An early diagnosis is not always simple, since minimal cutaneous manifestations are often the only signs in the early stage.

At physical examination, the classic early manifestation is a bulla filled with serous fluid and erythema and hemorrhagic and gangrenous lesions come into view.

Late stage is characterized by the presence of numerous large hemorrhagic bullae, necrotic tissue, crepitus, fluctuance and altered sensity, and motor deficits.

Considering the leukocyte count of each patient, we remark that 6 of the patients included in this review show normal white blood cell count. The remaining 3 patients have values ranging between 16.15 × 10^3^/*μ*l and 25 × 10^3^/*μ*l.

We can explain the normal leukocyte count of the majority of patients by immunosuppression and use of corticosteroid or NSAIDs, which can be responsible of immune unresponsiveness or anergy. Moreover, NSAIDs and corticosteroids might play an important role in NSTIs onset. They inhibit inflammatory cascade acting on TFN*α* and other cytokines. Furthermore, they are inhibitors of neutrophil granulocyte chemotaxis and phagocytosis by blocking the lipoxygenase pathway. They also decrease leukotriene production by leucocytes, stopping its proinflammatory role. Finally, they mask the progression of disease by suppressing fever and pain through inhibition of prostaglandin synthesis.

## 4. Conclusion

In our case, the patient was immunosuppressed following oral treatment with corticosteroids and Diclofenac. The injection administrated for the upper respiratory tract infection was decisive for NSTIs onset, because a plausible necrosis in the site of injection could have been infected by bacterial translocation inserting the needle or bacterial implantation after a transient bacteremia as an effect of respiratory phlogosis. The delayed clinical presentation could be strictly correlated to the administered therapy and to her state of immunosuppression [3, 4], since she showed up to hospital at a very late stage after the injection.

From this review, it is evident that a prompt treatment represents an essential condition to improve the overall prognosis and mortality. Patients with NSTIs occurred after intramuscular injections have a worse clinical outcome than patients with different entry routes. In immunosuppressed patients or receiving corticosteroids or NSAIDs, an appropriate injection technique is a crucial approach to prevent NSTIs. As a final point, particular attention should be paid to sharp pain onset in the injection site as an early sign.
